# Analysis of *Nanoarchaeum equitans *genome and proteome composition: indications for hyperthermophilic and parasitic adaptation

**DOI:** 10.1186/1471-2164-7-186

**Published:** 2006-07-25

**Authors:** Sabyasachi Das, Sandip Paul, Sumit K Bag, Chitra Dutta

**Affiliations:** 1 Bioinformatics Centre, Indian Institute of Chemical Biology, Kolkata–700032, India; 2 Human Genetics & Genomics Division, Indian Institute of Chemical Biology, Kolkata–700032, India

## Abstract

**Background:**

*Nanoarchaeum equitans*, the only known hyperthermophilic archaeon exhibiting parasitic life style, has raised some new questions about the evolution of the Archaea and provided a model of choice to study the genome landmarks correlated with thermo-parasitic adaptation. In this context, we have analyzed the genome and proteome composition of *N. equitans *and compared the same with those of other mesophiles, hyperthermophiles and obligatory host-associated organisms.

**Results:**

Analysis of nucleotide, codon and amino acid usage patterns in *N. equitans *indicates the presence of distinct selective constraints, probably due to its adaptation to a thermo-parasitic life-style. Among the conspicuous characteristics featuring its hyperthermophilic adaptation are overrepresentation of purine bases in protein coding sequences, higher GC-content in tRNA/rRNA sequences, distinct synonymous codon usage, enhanced usage of aromatic and positively charged residues, and decreased frequencies of polar uncharged residues, as compared to those in mesophilic organisms. Positively charged amino acid residues are relatively abundant in the encoded gene-products of *N. equitans *and other hyperthermophiles, which is reflected in their isoelectric point distribution. Pairwise comparison of 105 orthologous protein sequences shows a strong bias towards replacement of uncharged polar residues of mesophilic proteins by Lys/Arg, Tyr and some hydrophobic residues in their Nanoarchaeal orthologs. The traits potentially attributable to the symbiotic/parasitic life-style of the organism include the presence of apparently weak translational selection in synonymous codon usage and a marked heterogeneity in membrane-associated proteins, which may be important for *N. equitans *to interact with the host and hence, may help the organism to adapt to the strictly host-associated life style. Despite being strictly host-dependent, *N. equitans *follows cost minimization hypothesis.

**Conclusion:**

The present study reveals that the genome and proteome composition of *N. equitans *are marked with the signatures of dual adaptation – one to high temperature and the other to obligatory parasitism. While the analysis of nucleotide/amino acid preferences in *N. equitans *offers an insight into the molecular strategies taken by the archaeon for thermo-parasitic adaptation, the comparative study of the compositional characteristics of mesophiles, hyperthermophiles and obligatory host-associated organisms demonstrates the generality of such strategies in the microbial world.

## Background

The hyperthermophilic archaeon *Nanoarchaeum equitans *is characterized by several intriguing features. It is the only known parasitic archaeon and for survival it must be in contact with the crenarchaeon host *Ignicoccus*. Its genome size is only 490 kb, representing the smallest microbial genome known to date, and yet it has the highest coding density, encoding for 536 genes [1]. Phylogenetic analyses suggested that this microbe is probably a derived, but genomically stable parasite diverged anciently from the archaeal lineage [2].

The genes for several vital metabolic pathways appear to be missing in *Nanoarchaeum *[1]. This could be due to two plausible reasons. *N. equitans *might represent an ancient species, and hence possesses a small genome. Alternatively, it might have gone through a process of genome reduction as a strategy of adaptation to the obligatory parasitic lifestyle, as observed in cases of many other parasitic/symbiotic organisms [1]. However, in obligatory intracellular bacteria, many genes involved in DNA recombination and repair along with the biosynthetic and metabolic genes are usually lost [3,4]. But *N. equitans *possesses most of the DNA repair enzymes and the complete genetic machinery necessary for transcription, translation and DNA replication. The complexity of its information processing systems and the simplicity of its metabolic apparatus, therefore, suggest the presence of an unanticipated world of organisms yet to be characterized.

Most of the obligatory symbiotic/parasitic bacteria are characterized by the presence of only one/two rRNA operons, a small number of genes for tRNA isoacceptors, slow growth rate and an overall AT-richness [5-7]. It has been proposed that at the beginning of the symbiotic or parasitic integration, the loss of the genes involved in DNA repair favored the bias toward A+T content of such genomes [3,4,8]. However, the presence of a full set of archaeal DNA repair and recombination enzymes in *N. equitans *[1] contradicts the established hypothesis regarding AT-richness of its genome. Evidences for apparently little or no translational selection in synonymous codon usage have been reported for most of the species with reduced genomes examined so far, such as *Borrelia burgdorferi *[9], *Buchnera aphidicola *[10], *Helicobacter pylori *[11], *Bartonella *[12], *Wigglesworthia *[13] and *Tropheryma whipplei *[14] etc. Some of these bacteria exhibit strong base compositional asymmetries between leading and lagging strands of replication. It was, therefore, of interest to investigate whether *N. equitans*, an archaeon, is also characterized by any of such typical traits of the reduced genomes.

The composition of *N. equitans *genome and proteome are expected to bear the signatures not only of parasitism, but also of hyperthermophilicity. Comparative analysis of complete genomes of several hyperthermophilic archaea and bacteria revealed that organisms adapted to high temperature require a coordinated set of evolutionary changes towards stability of mRNA, codon-anticodon interactions [15], increased thermostability of encoded proteins by van der Waals interactions [16], larger number of residues in the alpha-helical conformation [17], enhanced secondary structure propensity [18], higher core hydrophobicity [19], additional networks of hydrogen bonds [20], increased ionic interactions [21], increased packing density [22], decreased length of surface loops [23] etc. With increase in growth temperature, microbial organisms tend to acquire base A and lose base C while keeping the contents of bases T and G relatively constant [24], and there is a clear link between a particular pattern of codon usage and the elevated growth temperature [25].

The current report presents an extensive study on the genome and proteome composition of *N. equitans*, along with a comparative analysis of the compositional characteristics of other mesophiles, hyperthermophiles and obligatory host-associated organisms. Only A+T -rich organisms were selected for analysis, so that the inter-species differences in nucleotide/amino acid usage patterns due to mutational bias could be minimized and any difference in such usage patterns among the mesophilic and hyperthermopilic organisms could be fairly attributed to their adaptation to the growth temperature. The study provides a more detailed view on the genome-wide strategies employed by *N. equitans *for adaptation to the hyperthermophilic environment and parasitic life style.

## Results

### Nucleotide preferences in protein-coding genes and structural RNA sequences

**Figure 1:**
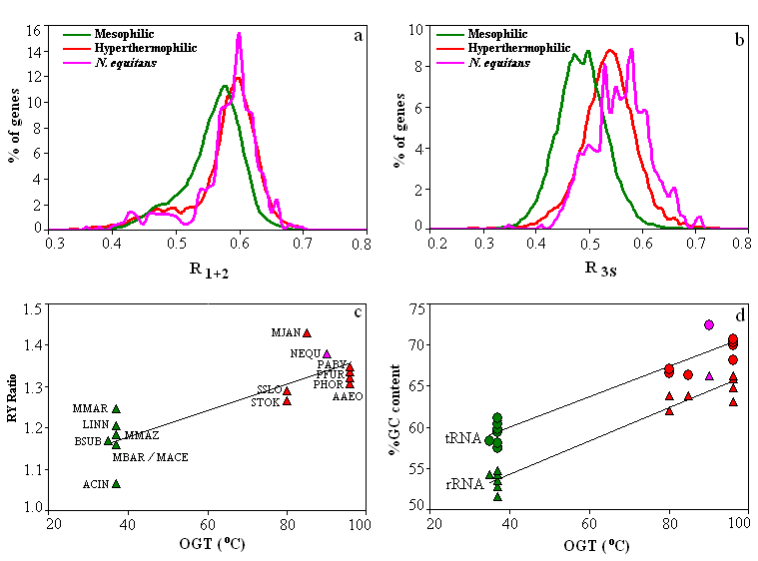
Distribution of genes on the basis of purine content at (a) nonsynonymous codon positions (R_1+2_); (b) synonymous codon positions (R_3S_) for seven mesophiles (Green line), seven hyperthermophiles (Red line), and *N. equitans *(Pink line). (c) Relationship between optimal growth temperature (OGT) and average Purine-pyrimidine ratio (RY ratio) of protein coding sequences. (d) Relationship between optimal growth temperature (OGT) and average GC-content of structural RNA sequences. The green, red and pink colors represent mesophiles, hyperthermophiles and *N. equitans *respectively. The filled circles for tRNAs and the filled triangles for rRNAs.

We have analyzed the annotated open reading frames (ORFs) and the tRNA and rRNA sequences separately in *N. equitans *and other organisms with a view to scrutinizing the trends in nucleotide selection and their relevance to the lifestyle of the organism. Table 1 presents the base composition of the ORFs and structural RNA sequences of the fifteen organisms under study. The frequency distributions of the ORFs of *N. equitans *and other hyperthermophiles exhibit a significant (p < 10^-7^) shift towards higher purine content as compared to those of the mesophiles for both non-synonymous and synonymous codon positions (Fig. 1a, b). As expected, there is a strong positive correlation (r = 0.89, p < 10^-4^) between the overall purine-pyrimidine ratio (R/Y) of ORFs and the optimal growth temperature (OGT). This suggests that the higher the OGT, the higher is the selection for purine nucleotides in coding sequences (Fig. 1c).

While the predicted ORFs of hyperthermophiles are characterized by overrepresentation of purine content, the structural RNA genes of *N. equitans *and other hyperthermophiles exhibit much higher GC-content than those of the mesophiles (Table 1). The GC-content of tRNA/rRNA genes exhibit a strong positive correlation (r = 0.98 and 0.96 at p < 10^-4 ^for rRNA and tRNA respectively) with the optimal growth temperature (OGT) (Fig. 1d). Similar observation has been reported earlier in thermophilic prokaryotes [26,27]. The higher GC-content of non-coding RNA sequences in *N. equitans *and other hyperthermophiles could be a strategy to facilitate the intramolecular stabilization of RNA secondary structure at elevated temperature. This notion is in agreement with earlier reports [28,29] demonstrating a significant correlation between the growth temperature and the GC-content of 16S rRNA, which is shown to be strongest in the double-stranded stem regions of the rRNA.

**Figure 2:**
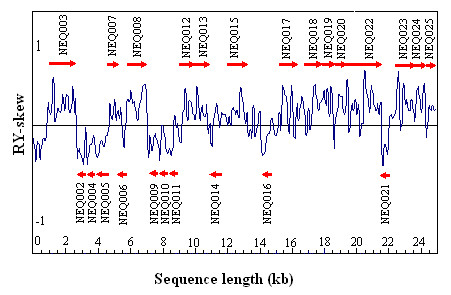
Plot of purine-pyrimidine (R-Y) skew along the first 25 kb of the genomic sequence of *N. equitans*. The locations of putative ORFs are shown as red arrows (upper arrows for ORFs present in direct strand; lower arrows for ORFs present in complementary strand).

In order to evaluate purine richness in protein coding sequences, we have calculated the Chargaff differences in nucleotide composition (a measure of purine-loading) for the entire genome. Like other hyperthermophilic prokaryotes [24], *N. equitans *strictly follows Szybalski's transcription direction rule in spite of its obligatory parasitic lifestyle. The purine-pyrimidine skew correlates strongly with the location of the ORFs in two strands (Fig. 2) and therefore, the ORFs residing in the direct strand as well as those in the complementary strand, in general, tend to be purine-rich. It was proposed earlier that the selection for purine-rich mRNA sequences in thermophilic organisms may minimize unnecessary RNA-RNA interactions and prevent double-strand RNA formation within the molecule [30]. The purine-loading in the coding sequences of *N. equitans*, therefore, may be attributed to its adaptation to high temperature. The GC-content of predicted ORFs or the purine content of structural RNA sequences does not exhibit any significant correlation with the optimal growth temperature of the organisms under study.

### Global analysis of proteome composition

**Figure 3:**
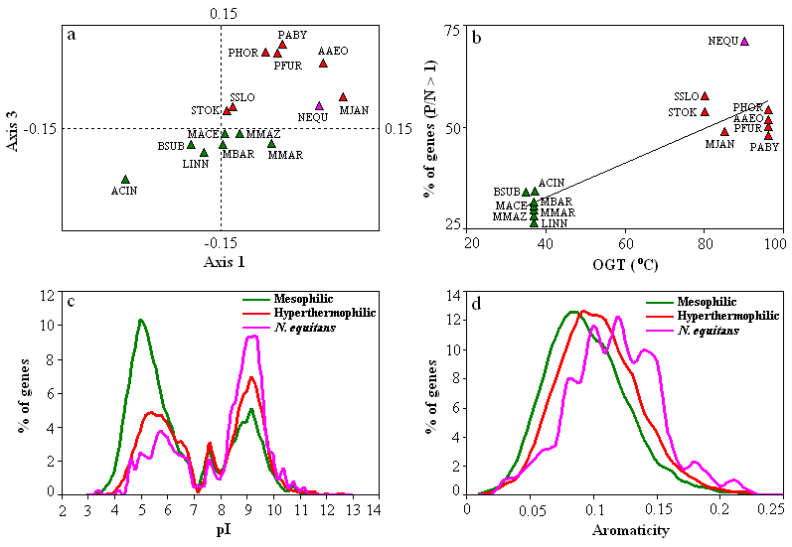
(a) Positions of seven mesophiles, seven hyperthermophiles and *N. equitans *on the plane defined by first and third axes generated from COA on amino acid usage of encoded proteins. (b) Relationship between optimal growth temperature (OGT) of the organisms and the number of genes having ratio of positively charged and negatively charged residues greater than one (P/N ratio > 1) in the encoded proteins of respective organisms. (c) Plot for the distribution of genes versus predicted isoelectric point (pI) of encoded proteins. (d) Distribution of genes on the basis of aromaticity of encoded proteins. Green color indicates mesophiles, red color indicates hyperthermophiles and pink color indicates *N. equitans*.

Correspondence analysis (COA) has been carried out on amino acid usage of the 35056 predicted gene-products of the fifteen organisms under study (Table 1) to find out the interproteomic variation in amino acid composition, if any. The analysis reveals that the mesophiles and hyperthermophiles are clearly segregated on positive and negative sides of axis 3, which represents 10.6% of total variation (Fig. 3a). Thus amino acid usage patterns follow distinct trends in hyperthermophilic (including *N. equitans*) and mesophilic organisms irrespective of the phylogenetic relatedness, supporting previous observations [15,31]. The encoded proteins in thermophiles are characterized by an increase in frequency of charged amino acid residues and a decrease in that of polar uncharged residues as compared to the mesophilic counterparts [32,33]. However, a discrepancy is noticeable in the overrepresentation of positively and negatively charged residues in hyperthermophilic proteins. The percentage of proteins having positively charged to negatively charged amino acid ratio (P/N) > 1 is significantly high in hyperthermophiles compared to mesophiles. A strong positive correlation (r = 0.88, p < 10^-5^) exists between the OGT of the organisms and the percentage of proteins having P/N ratio greater than one in the respective proteomes (Fig. 3b).

Overrepresentation of positively charged residues in the gene-products of *N. equitans *and other hyperthermophiles is also apparent from Fig. 3c, which shows the predicted isoelectric point (pI) distribution of the proteins of the fifteen organisms under study. A bimodal distribution of isoelectric points is observed with an acidic peak at pI range of 5.0–5.5 and a basic peak at ~9.5. For the mesophiles, the acidic peak is much larger than the basic peak, while the reverse is the case for hyperthermophiles. For *N. equitans*,the number of basic proteins is even appreciably higher than that observed with other hyperthermophiles (Fig. 3c). These results suggest that the hyperthermophilic proteomes are characterized by a relative predominance of basic proteins. In addition to the relative abundance of positively charged residues, the frequency distribution of the encoded proteins of *N. equitans *and other hyperthermophiles is also shifted significantly (p < 10^-7^) towards higher aromaticity, as compared to that of the mesophiles (Fig. 3d).

### Comparison with mesophilic orthologs

The pairwise comparison of 105 protein sequences from *N. equitans *and their homologs from seven mesophilic organisms under study shows that there has been a significant increase in frequencies of positively charged residues (but not of overall negatively charged residues) in *N. equitans *proteins (Table 2). Among negatively charged residues, Glutamic acid but not Aspartic acid, is used with increased frequency in hyperthermophiles. Furthermore, there are significant increases in aromaticity and average hydrophobicity (calculated using Sweet and Eisenberg scale) [34] and a decrease in the usage of polar uncharged amino acid residues (Ser, Thr, Gln and Asn) in the *N. equitans *gene-products (Table 2). Previous studies on thermo-adapted organisms reported a trend for increase in number of overall charged residues [32,33]. But the present analysis clearly indicates that among the charged residues, frequencies of the positively charged residues increase significantly.

In order to get a better insight into such trends, we have determined the frequencies of all possible amino acid replacements (*i.e*., [20 × 19]/2 = 190 possible pairs of replacements) between the orthologous sequences in the direction of mesophiles to *N. equitans *[see Additional file 1]. Table 3 shows the ratios of the number of observed forward and reverse replacements for each pair of residues in the direction from mesophiles to *N. equitans*. There are 36 pairs of amino acids (20% of all pairs) that have a significant directional replacement bias (p < 0.01) and they contribute 6,347 of the 16,614 observed replacements (38% of the replacements). Several of these frequently observed amino acid replacements (i.e., Leu → Ile, Val → Leu, Met → Ile etc.) are conservative and have already been reported [32,35]. It is worth mentioning that some of the apparently high values of the ratios of replacements may not have much impact on thermal adaptation of proteins, as the actual number of replacements is too small. For example, for Cys → Leu, the high value (= 7) of the forward to reverse replacement ratio may not have any structural significance as the number of replacements in the forward (Cys → Leu) and reverse (Leu → Cys) directions are only 14 and 2 respectively [see Additional file 1]. The analysis shows that in *N. equitans*,the uncharged polar residues of mesophilic proteins have undergone massive replacement by positively charged residues (especially Lys), aromatic residues (especially Tyr) and some hydrophobic residues (*e.g*. Ile, Leu etc.), but the replacements in the reverse direction are not so frequent. The highly biased directionality of such amino acid replacements suggests that the favored residues in *N. equitans *proteins may have significant contribution to enhancement and/or maintenance of thermostability.

### Surface charge distribution

**Figure 4:**
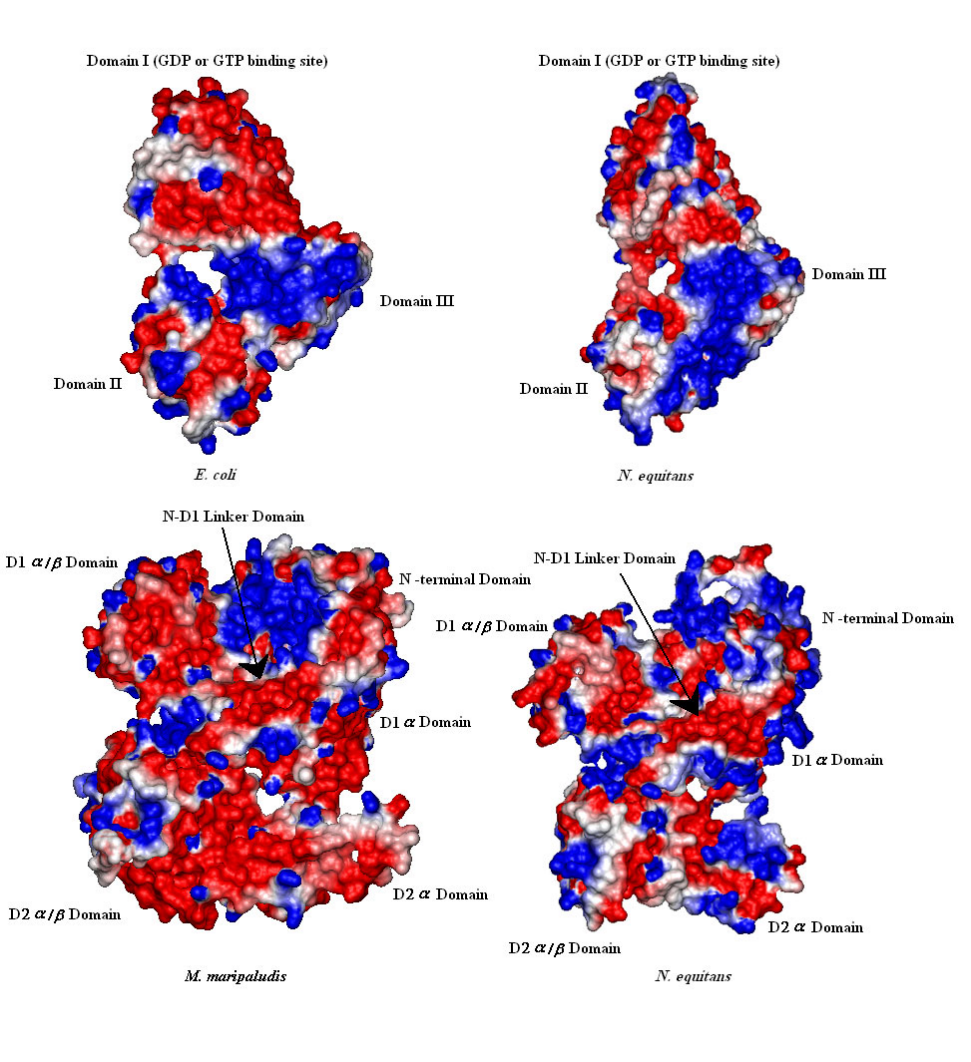
Surface charge distributions for the Elongation factor Tu (EF- Tu) proteins of *E. coli *(upper-left) and *N. equitans *(upper-right) and the Cell division cycle family protein from *M. maripaludis *(lower-left) and *N. equitans *(lower-right). Basic region is indicated by blue color, acidic region is indicated by red color and neutral region is indicated by white color.

In order to examine the structural implications, if any, of higher usage of the positively charged residues in *N. equitans *proteins, appropriate representatives of orthologous pairs have been selected for homology modeling. Modeled structures have been generated for the elongation factor Tu (EF- Tu) protein of *N. equitans *(NEQ082), along with the cell division cycle (CDC) family protein from *N. equitans *(NEQ475) and *M. maripaludis *(MMP0176). The surface charge has been determined for each protein using Coulomb charge calculation. The comparison between *N. equitans *EF- Tu and *E. coli *EF- Tu [36] and between the CDC proteins from *N. equitans *(NEQ475) and *M. maripaludis *(MMP0176) reveals a marked increase in positive charge in the surfaces of the *N. equitans *proteins as compared to their mesophilic counterparts (Fig. 4). The overall surface charge of *N. equitans *EF-Tu is -2, while that of its *E. coli *homolog is -16. The *N. equitans *CDC exhibits a surface charge of -2, while that for its *M. maripaludis *homolog is -20. These findings are consistent with the higher isoelectric points and higher frequencies of positively charged residues in *N. equitans *homologs. In EF-Tu of *N. equitans *(pI = 7.85), the cumulative frequency of Arg and Lys is 13.2%, whereas in *E. coli *EF-Tu (pI = 5.30), it is 11.8%. The frequencies of positively charged residues for the CDC homologs NEQ475 (pI = 7.55) and MMP0176 (pI = 5.19) are 16.8% and 14.5% respectively. Higher usage of positively charged residues in *N. equitans *proteins results in an increase in positive charge on their surfaces as compared to their mesophilic homologs.

### Intraproteomic variations in amino acid usage

**Figure 5:**
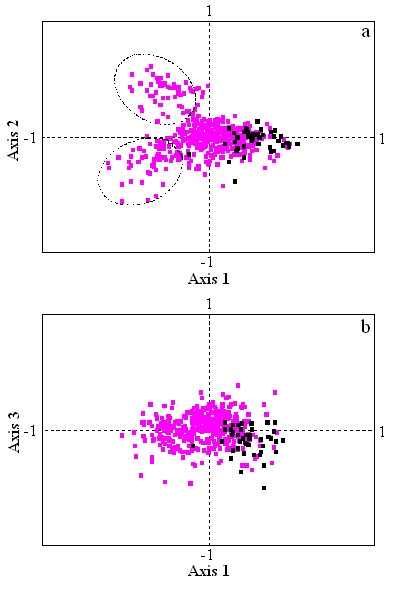
COA on amino acid usage for encoded proteins of *N. equitans*. (a) Position of genes on the plane defined by axis 1 and axis 2. The upper and lower clusters of genes mainly encoding membrane-associated proteins are marked by dashed-line ovals. (b) Position of genes on the plane defined by axis 1 and axis 3. Black quadrangle and pink quadrangle represent highly expressed and other genes respectively.

To understand the trends in amino acid usage of the encoded proteins in *N. equitans*, COA has been performed on the amino acid composition. Mean aromaticity, hydropathy, aliphatic index and gene expressivity are the major sources of intra-proteomic variations in *N. equitans*, as indicated by the COA on amino acid usage of the encoded proteins of 487 ORFs (Table 4). There are two distinct clusters of genes along the 2nd major axis near the left end of axis 1 (Fig. 5a). The proteins encoded by these two clusters of genes are mainly cell envelope or secreted proteins, which comprise about 15 % of the total predicted proteins under study. The encoded proteins of the upper cluster (UC) genes are significantly over represented (p < 0.001) by Phe, Leu, Ile, Met and Ala, whereas Tyr, Cys, Gln, Thr, Asn, Lys, Asp and Glu are present in significantly higher amounts (p < 0.001) in the lower cluster (LC) proteins (Table 5). These two groups of proteins also differ in their predicted secondary structures as well as in the content of potential disordered structures. For the members of the UC, propensities for the formation of alpha helix structure (mean value 47.77) are much higher than for the formation of beta sheet (mean value 17.33). On the contrary, the proteins encoded by the LC genes have higher propensities for the formation of beta sheet (mean value 28.70) than alpha helix (mean value 19.74) structure. Furthermore, the proteins encoded by the UC genes have significantly lower propensities for the formation of random coil (mean value 34.90) and putatively have 5–7 transmembrane domains, while the members of LC, in general, show higher propensities for the formation of random coil (mean value 51.56) and are predicted to have only 1–2 transmembrane domains [see Additional file 3]. Disordered regions in proteins can be predicted by the lack of regular secondary structures, whereas ordered regions (often termed globular) typically contain regular secondary structures packed into a compact globule [37,38]. In *N. equitans*, the probable coil forming regions are significantly higher in LC proteins (Table 5); hence, disordered structures are more commonly found in the proteins of LC than in the proteins comprising the UC.

Another important source of intra-proteomic variations in amino acid usage is gene expressivity, as indicated by the presence of potential highly expressed genes near the positive extreme of axis 1 and at the negative extremes of axis 3 (Fig. 5b). Both axes exhibit significant correlation with CAI values of the genes (Table 4). The significant positive correlation of MMW with axis 3 and the negative correlation between axis 1 and aromaticity (Table 4) suggest that the potential highly expressed genes in *N. equitans *have a tendency to avoid the heavier residues including the aromatic ones (Table 5). *N. equitans *is a strictly host-adapted microorganism, which can exploit the cellular machinery of host organisms for its own survival. It is therefore interesting to note that it follows the cost minimization hypothesis [39], which claims that highly expressed genes tend to use small and energetically less expensive amino acids in their encoded proteins.

### Evaluation of hyperthermophilic signature on synonymous codon usage

**Figure 6:**
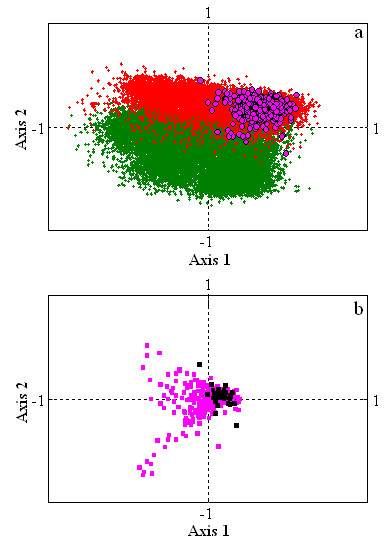
Position of genes along the first two principal axes generated by COA on RSCU values of (a) 35056 genes from 15 microbial genomes under study (hyperthermophilic: red dots, mesophilic: green dots, pink circle: *N. equitans*); (b) 487 genes from *N. equitans *(black quadrangle: potential highly expressed genes, pink quadrangle: the other genes).

In an attempt to examine whether the pattern of synonymous codon usage in *N. equitans *follows the hyperthermophilic signature, COA has been applied on Relative Synonymous Codon Usage (RSCU) of 35056 predicted ORFs of the fifteen microbial genomes under study (enlisted in Table 1). The axis 1- axis 2 plot of the COA on RSCU values exhibits two distinct clusters, the mesophilic and hyperthermophilic (*N. equitans *being a subset of the latter) genes being segregated along the second axis with little overlap between them (Fig. 6a). This is in accordance with the studies made earlier by Lynn et al. [25]. Axis 1 (representing 16.2 % of total variation) values are highly correlated to the GC_3S _values (r = -0.93, p < 10^-7^), while axis 2 (representing 13.7 % of total variation) values exhibit a significant positive correlation with R_3S _(purine content in synonymous third codon position) of the genes (r = 0.54, p < 10^-7^). These observations indicate that the pattern of synonymous codon usage in *N. equitans*,as with other hyperthermophiles, is different from that observed in the mesophilic microbial organisms, where the usage of purine in third codon positions is comparatively lower.

### Intragenomic variation in synonymous codon usage

To understand the sources of intragenomic variation in codon usage of *N. equitans *we have applied a COA on RSCU of its 487 ORFs. Axis 1 exhibits significant positive correlation with the CAI values of the genes and also exhibits slight but significant positive correlation with A_3S _(Table 4). Most of the potential highly expressed genes including ribosomal proteins are clustered at the positive extreme of axis 1 (Fig. 6b). In COA on absolute frequencies of synonymous codon usage also, the first major axis (representing 11.5 % of total variation) exhibits significant correlation with CAI values (r = -0.52, p < 0.0001) and the putative highly expressed genes are clustered on the negative side of that axis. These observations suggest that the major trend in synonymous codon usage is gene expressivity. Usage of 16 codons increases significantly (p < 0.05) in potential highly expressed genes, most of which prefer to use A-ending or C-ending synonymous codons [see Additional file 2]. However the frequencies of G-ending codons or U-ending codons either remain almost constant in potential highly and lowly expressed genes (except AGG codon for Arg, UGU for Cys and GGU for Gly), or show a marked fall in potential highly expressed genes. Preference for C-ending codons in highly expressed genes against the genome-wide mutational bias is probably a consequence of translational selection [40]. No known parameter of codon usage, base or amino acid composition is found to have significant correlation with the position of sequences on axis 2 (Table 4). But interestingly enough, there is a divergence in the distribution of few genes along axis 2 near the negative extreme of axis 1. Careful examination reveals that this is only due to the differential usage of four rare synonymous codons (CGN of Arg) in *N. equitans*.

The synonymous codon bias in the potential highly expressed genes of *N. equitans *may not be very strong, as axis 1 of the COA on RSCU describes a rather small amount of total variation – a situation encountered in most of the obligatory symbiotic/parasitic microbial organisms (Table 6). It is worth mentioning at this point that the strictly host-associated microorganisms are usually characterized by a reduced genome, presence of only one or two rRNA operons, small number of tRNA genes, long generation time, overall AT-richness, and apparently weak translational selection for synonymous codon usage [11,13]. The genome of *N. equitans *is also characterized by massive reduction in size as well as decrease in overall GC-content, as compared to the free-living hyperthermophilic archaea. It encodes only a limited number of tRNAs (38 identified tRNAs) and single copies of 5S, 16S and 23S rRNA. The existence of a relatively poor translational selection in *N. equitans *is, therefore, quite consistent with its parasitic lifestyle.

## Discussion

The present analysis indicates that the dual adaptation of *N. equitans *to high temperature and to an obligate parasitism has imposed selective constraints on nucleotide usage at synonymous and nonsynonymous codon positions, modulating thereby its genome/proteome composition. Thermal adaptation involves overrepresentation of purine bases in protein coding sequences, higher GC-content of the structural RNA genes, enhanced usage of positively charged residues, higher frequencies of aromatic residues, decrease in polar uncharged residues in the encoded protein etc., while parasitic adaptation is reflected in the extreme genome reduction, presence of weak translational selection for synonymous codon usage, limited number of tRNAs and rRNAs, large heterogeneity in membrane associated proteins and so on.

One of the most exciting observations is the significant increase in the usage of positively charged amino acid residues in encoded proteins of *N. equitans *and other hyperthermophiles compared to that in mesophilic organisms. A strong positive correlation between the optimal growth temperature of the organisms and the percentage of proteins with P/N ratio > 1 in the respective proteomes (Fig. 3b), relatively basic nature of the proteomes of *N. equitans *and other hyperthermophiles, as depicted by isoelectric point distribution (Fig. 3c) and bias in the replacement of uncharged polar residues of mesophilic proteins by positively charged residues (mainly Lys) in the *N. equitans *orthologs (Table 3) – all point towards a strong preference for positively charged amino acids in the gene-products of hyperthermophiles. In parallel, there has also been an increase in aromatic residues (especially Tyr) in encoded proteins of *N. equitans *and other hyperthermophiles (Fig. 3d; Table 3). Greater involvement of positively charged residues at or near protein surfaces may increase the probability of salt bridge formation with negatively charged residues, while simultaneous increase in aromatic residues may strengthen the cation-π interaction. When a cationic side chain comes near an aromatic side chain within a protein, the geometry is known to be biased towards one that would experience a favorable cation-π interaction [41]. It was suggested earlier that both salt-bridge and cation-π interactions may play important roles in thermostability [42]. Tyrosine, by itself, may also contribute to protein thermostability [43]. Selective increase in Lys/Arg and aromatic residues in *N. equitans *may, therefore, be a strategy of survival at high temperature. A recent study on atomic simulation has revealed that among the charged residues, Lys has much greater number of accessible rotamers than Arg and may entropically stabilize the folded states of proteins [44].

Marked reduction in the frequencies of uncharged polar residues may also contribute to thermostability by avoiding the deamination and backbone cleavages involving Asn and Gln, which can be catalyzed by Ser and Thr [45,46]. According to the Sweet and Eisenberg scale [34], there is a significant increase in average hydrophobicity in the encoded proteins of *N. equitans*, as compared to their mesophilic orthologs. This may also be a part of the measures taken for environmental adaptation. The Kyte-Doolittle scale [47], however, did not indicate any significant difference in average hydrophobicity between these two groups of proteins. It was suggested earlier by Haney et al. [32] that some of the established hydrophobicity scales are strongly correlated to the differences between the proteins of mesophiles and thermophiles, whereas others are not. Replacement of the uncharged polar residues of mesophilic proteins by more hydrophobic residues in *N. equitans *orthologs may lead to an increase in the extent of the hydrophobic core and hence to a decrease in the solvent accessible surface area of the protein. Therefore the stability of *N. equitans *proteins in extremely high temperatures is apparently provided by significant modifications in their sequences toward enrichment of certain residues.

It is interesting to note that unlike mesophilic organisms, in *N. equitans *and other hyperthermophiles, there is a markedly differential selection for nucleotide usage in protein coding and structural RNA sequences. Both nonsynonymous and synonymous codon positions of their coding sequences are purine rich, and this has two probable consequences. Firstly, due to purine richness of protein-coding sequences, the organisms may minimize unnecessary RNA-RNA interactions and prevent the double-stranded RNA formation within the molecule [30]. Secondly, the higher purine content in nonsynonymous codon position has good correlation with the increased frequencies of certain residues in the encoded proteins. These may help the organism to adapt in high temperature. In contrast, the structural RNA sequences (tRNAs and rRNAs) of these organisms are characterized by significantly higher GC-content and hence by an increased number of hydrogen bonds, which may facilitate intramolecular stabilization at elevated temperature [29,48]. Thus, in *N. equitans*, selection for the prevalence of purine bases in ORFs and the GC- richness of non-coding RNA sequences may also be the consequence of its hyperthermophilic adaptation.

Recent studies on several microbial genomes indicate a close connection between synonymous codon usage bias, tRNA abundance, number of rRNA operons, optimal generation time and genome size [6,7,49]. Most of the species with reduced genomes are host-associated microorganisms, characterized by the presence of only one or two rRNA operons, a small number of tRNA genes, long generation time and overall AT-richness. Evidences for apparently little translational selection have been reported in most of these organisms. *N. equitans*, which is an archaeal parasite with the smallest genome known so far, encodes only a limited number of tRNAs (38 identified tRNAs) and single copies of 5S, 16S and 23S rRNA. Although *N. equitans *is a hyperthermophilic archaeon, evidence for a relatively poor translational selection for synonymous codon usage is consistent with the earlier observations on several bacteria adapted to strictly host-associated lifestyle. Furthermore, the synonymous codon usage pattern of *N. equitans *forms a subset of the patterns observed typically in hyperthermophilic microbial species and is quite distinct from the patterns of the mesophilic organisms (Fig. 5a). Synonymous codon usage in Nanoarchaeal genes, therefore, reveals a dual adaptation to obligatory parasitism and hyperthermophilicity.

As demonstrated by the COA on amino acid usage, probable membrane associated proteins exhibit two different clusters (Fig. 4a). The amino acid usage profile and the predicted secondary structures of the members of these two clusters are quite distinct from one another (Table 5). Most of the variations in cell-surface proteins may be potentially important for *N. equitans *to interact with the *Ignicoccus *host and probably evolved during the course of parasitic and/or thermal adaptation. It is also important to note that in spite of its obligatory parasitic lifestyle, there is a tendency to follow the cost-minimization hypothesis at lower level as proteins encoded by highly expressed genes are preferentially constructed with some smaller and energetically less expensive amino acids. Existence of cost minimization effect in host-associated organisms might be due to a genome-level adaptation to utilize less expensive and small residues from the host in the highly expressed genes [12,14,50]. This might have an evolutionary advantage to minimize host energy exhaustion for maintaining continued association and the chance of elimination by the host.

Hyperthermophilic organisms, in general, have comparatively smaller genome than the mesophilic organisms. It might be advantageous in hyperthermophiles to maintain multiple copies of chromosomes per cell due to a probable need of a reserve supply of intact chromosome to compensate for the greater chance of DNA double strand breaks at high temperature [51,52]. Furthermore, the faster replication of small genome is likely to be more favorable in the environment having temperatures near to or above 100°C [52]. Many microbial organisms living in close association with other organisms in an obligate symbiotic or parasitic relationship also experienced a reduction in genome size with respect to their free-living ancestors. The *N. equitans *genome lacks the genes for central metabolism, primary biosynthesis and bioenergetic apparatus [1], which are expected to be present in the common archaeal ancestor. In contrast to mesophilic organisms, it possesses the simplest functional protein folding system – the genome contains only single copies of homologues of prefoldin α- and β-subunits, Hsp60 and sHsp [52]. Unlike other obligate symbiotic/parasitic organisms, *N. equitans *has well-organized DNA repair mechanism with a full set of archaeal DNA repair and recombination enzymes [1]. Furthermore, despite the small genome size, it devotes a large amount of coding capacity for surface-associated proteins, suggesting that the interaction with its host may play a major part in the parasitic adaptation of the organism. Hence, it can be inferred that the unusual genome reduction and genome composition in *N. equitans *are the consequences of both hyperthermophilic and parasitic adaptation and during the coevolutionary process with *Ignicoccus *host, *N. equitans *may have experienced a dramatic decrease of genome size, retaining only the essential genes for its thermo-parasitic lifestyle.

## Conclusion

Comprehensive analysis on the *N. equitans *genome along with its comparison to other mesophiles, hyperthermophiles and host-associated organisms allowed us to understand how the dual adaptation of *N. equitans *to high temperature and to an obligate parasitism can influence the nucleotide usage at synonymous and nonsynonymous codon positions, modulating thereby its genome/proteome composition. Thermal adaptation involves overrepresentation of purine bases in protein coding sequences, higher GC-content of the structural RNAs, enhanced usage of positively charged residues and aromatic residues, decrease in polar uncharged residues in the encoded protein and so on, while the parasitic adaptation is reflected in the extreme genome reduction, presence of weak translational selection for synonymous codon usage, large heterogeneity in membrane associated proteins etc. Our findings not only offer an insight into the mechanisms of genomic adaptation of *N. equitans *to high temperature and parasitism, but also evaluate the generality of such mechanisms in the microbial world.

## Methods

### Sequence retrieval

All predicted protein coding sequences and the sequences of structural RNAs (tRNAs and rRNAs) of *Nanoarchaeum equitans *Kin4-M were extracted from NCBI GenBank (Version 145.0) [53]. To understand temperature related traits, we compiled sequences of predicted protein coding genes and structural RNAs from seven hyperthermophilic and seven mesophilic microbial organisms from GenBank [53]. For comparison purpose, the selection of these completely sequenced microbial organisms was based on the close approximation in genomic GC-content of *N. equitans *(i.e. all organisms under study are relatively AT-rich) to minimize the GC-compositional effect on codon as well as on amino acid usage. To compare with other host-associated organisms, sequences from seven obligatory parasitic/symbiotic microorganisms were also retrieved. In order to reduce sampling errors, the annotated genes with less than 100 codons were excluded from the analysis. The presumed duplicates, genes for transposase and integrase, and the genes with internal stop codons and/or untranslatable codons were also excluded.

### Base compositional analysis

To find out the extent of base compositional bias, nucleotide frequency at all three codon positions were calculated for protein coding sequences. The purine content at nonsynonymous codon position (R_1+2_) and synonymous third codon position (R_3S_) were calculated for each coding sequences of seven mesophilic, seven hyperthermophilic organisms and *N. equitans*. Purine-pyrimidine skew was performed using sliding windows of 0.1 kb on the genomic sequence of *N. equitans*. The GC content and also purine content of the structural RNA sequences were calculated for *N. equitans*, seven hyperthermophilic and seven mesophilic organisms under study.

### Correspondence Analysis on synonymous codon and amino acid usage

Correspondence Analysis (COA) was performed using the program CODONW 1.4.2 [54] to identify the major factors influencing the variation in relative synonymous codon usage (RSCU) and amino acid frequencies. COA was also carried out on absolute frequencies of synonymous codons in order to avoid introducing other biases [55]. These analyses generate a series of orthogonal axes to identify trends that explain the variation within a dataset, with each subsequent axis explaining a decreasing amount of the variation.

### Amino acid exchange bias with orthologous sequences

Orthologous sequences between *N. equitans *and seven mesophilic organisms under study were taken using the BlastP program [56]. Orthologs were defined as those with more than or equal to 60% similarities and less than 20% difference in length. The amino acid sequences of 105 orthologous genes were aligned using the pairwise alignment program (ClustalW) and the amino acid replacements were obtained in the form of a matrix, using a program developed in-house in Visual Basic. For a given pair of amino acids, the "forward" direction exhibited the more common of the two replacements in the conversion of mesophilic proteins to *N. equitans *proteins. To assess the significance of the directional bias, if any, replacement values were compared by 2 × 2 contingency tables having 1 degree of freedom. For each pair of replacements, the first and second rows of the contingency table represented the number of replacements from one particular residue (say, i) to another (say, j) of the pair and the total count of the remaining replacements (say, k) from the residue i (where k ≠ j) respectively.

### Prediction of secondary structure, transmembrane domain and protein disorder

The prediction of protein secondary structure was performed using GOR IV algorithm [57] and the disordered regions within proteins were predicted using GlobPlot [38]. SMART [58] and TMHMM2.0 [59] available at ExPASy Proteomics Server [60] were used to detect the proteins likely to secreted in or localized to the cell surface.

### Indices used to identify the trends in codon and amino acid usage

Indices like total number of occurrence of each codon, RSCU [61], codon adaptive index (CAI) [61], amino acids frequencies, average hydrophobicity (Gravy score) [34,47], aromaticity [62], aliphatic index [63] and mean molecular weight (MMW) of protein coding sequences were calculated to find out the factors influencing codon and amino acid usage. The CAI was calculated for *N. equitans *genes with respect to the RSCU values of the genes for ribosomal proteins (≥ 100 aa). The isoelectric point (pI) of each predicted proteins were calculated using Expasy proteomics server [60].

### Homology modeling

Modeled structures were generated for the elongation factor Tu (EF- Tu) of *N. equitans *and the cell division cycle family protein from both *N. equitans *and *M. maripaludis *(NEQ475 in *N. equitans *and MMP0176 in *M. maripaludis*) by using the First Approach Mode at the Swiss-Model protein structure homology modeling server [64]. The surface charge distributions were mapped onto the predicted surface using the program MOLMOL [65]. Total surface charge was calculated using Biomolecule module in Insight II workstation. Comparisons were made between *N. equitans *EF- Tu and *E. coli *EF- Tu (Blast P value 1e^-45^) and between the CDC proteins from *N. equitans *(NEQ475) and *M. maripaludis *(MMP0176) (Blast P value 0.0).

## Abbreviations

COA, Correspondence analysis; CAI, Codon adaptation index; OGT, Optimal growth temperature; ORF, Open Reading Frame; GC_3S_, GC-content at synonymous codon position; R_1+2_, Purine content at first and second codon positions; R_3S_, Purine content at synonymous codon position; MMW, Mean molecular weight; RSCU, Relative synonymous codon usage.

## Authors' contributions

SD and SP made substantial contributions to the conception of the study, devised the overall strategy, performed genome sequence analysis and drafted the manuscript. SKB developed relevant programs for sequence analysis and performed sequence alignment. CD participated in the design and coordination of the study and revised the manuscript critically for important intellectual content. All authors read and approved the final manuscript.

## Supplementary Material

Additional file 1 Amino acid replacement values between *N. equitans *proteins and their mesophilic orthologs. Click here for file

Additional file 2 Synonymous codon usage in *N. equitans* Click here for file

Additional file 3 TMHMM plot of the probable membrane associated proteins encoded by genes taken from upper and lower clusters generated by COA on amino acid usage for *N. equitans *genome. The plots of the left panel are the representatives of upper cluster and those in the right panels are the representatives of lower cluster. Red, blue and pink colours indicate the transmembrane, outside and inside regions of the protein respectively. Click here for file

## Figures and Tables

**Table 1:** General features of the *N. equitans *genome and 14 other microbial genomes under study

	Organism	Accession No. (GenBank)	Size (Mb)	Optimal growth temp °C	ORFs under study	GC- content (%)			
						
						Genome	ORFs	rRNAs	tRNAs
Hyperthermophilic	*Aquifex aeolicus*	AE000657	1.59	96	1485	43.3	43.7	64.8	68.4
	*Methanocaldococcus jannaschii*	L77117	1.74	85	1534	31.3	32.0	63.8	66.5
	*Nanoarchaeum equitans*	AE017199	0.49	90	487	31.5	31.2	66.2	72.5
	*Pyrococcus abyssi*	AL096836	1.77	96	1686	44.6	45.2	65.9	70.5
	*Pyrococcus furiosus*	AE009950	1.91	96	1845	40.7	41.2	66.3	70.5
	*Pyrococcus horikoshii*	BA000001	1.74	96	1717	41.8	42.3	63.2	70.7
	*Sulfolobus solfataricus*	AE006641	2.99	80	2741	35.7	36.5	62.1	67.3
	*Sulfolobus tokodaii*	BA000023	2.69	80	2558	32.7	33.6	63.8	67.4

Mesophilic	*Acinetobacter sp*.	CR543861	3.60	37	3020	40.4	41.3	51.7	58.1
	*Bacillus subtilis*	AL009126	4.21	35	3615	42.0	43.9	54.2	58.5
	*Listeria innocua*	AL592022	3.09	37	2677	37.0	37.8	52.8	58.23
	*Methanococcus maripaludis*	BX950229	1.66	37	1541	33.1	34.1	54.2	61.2
	*Methanosarcina acetivorans*	AE010299	5.75	37	3958	42.6	45.4	54.5	60.5
	*Methanosarcina barkeri*	CP000099	4.87	37	3206	39.2	42.4	53.6	59.5
	*Methanosarcina mazei*	AE008384	4.10	37	2986	41.4	44.4	54.2	60.7

**Table 2:** Differences between various indices of *N. equitans *proteins and theirmesophilic orthologs

Indices	*N. equitans *proteins	Mesophilic proteins	p
		
	Mean	Mean	
Average Hydrophobicity (Sweet & Eisenberg)	0.015	-0.056	1.7 × 10^-11^
Average Hydrophobicity (Kyte & Doolittle)	-0.349	-0.313	0.32061
Aromaticity Index	0.088	0.067	4.2 × 10^-9^
P/N Ratio	1.391	1.135	2.1 × 10^-3^
Positively Charged Residues (%)	17.1	14.5	3.0 × 10^-6^
Negatively Charged Residues (%)	13.7	13.3	0.31684
Isoelectric Point	8.332	7.158	1.4 × 10^-5^
Polar Uncharged Residues (%)	13.7	16.9	1.1 × 10^-13^

**Table 3:** Trends in amino acid replacements in *N. equitans *proteins and their mesophilic orthologs

	*N. equitans *amino acid																				
		Arg	Lys	Glu	Asp	Trp	Ile	Pro	Leu	Val	Phe	Tyr	Met	His	Ala	Gly	Cys	Thr	Asn	Gln	Ser
		
Mesophilic homologs amino acid	Ser	1.34	2.83*	2.47*	2.02	-	3.07*	2.65	2.30	2.43	3.33	2.55	2.44	0.81	1.35	1.13	0.14+	1.04	1.57	1.64	1.00
	Gln	1.52	1.56*	1.03	0.75	1.50	3.86*	2.18	2.13	0.74	1.00	2.60	1.21	1.20	0.72	0.55	0.00	0.29+	1.17	1.00	
	Asn	1.02	1.67*	0.87	0.96	2.00	1.71	0.91	1.14	0.65	0.54	3.00*	2.00	1.13	0.54	0.78	0.67	0.77	1.00		
	Thr	1.33	2.38*	1.91	1.00	4.00	2.23*	2.35	2.72*	1.47	1.45	5.00*	0.76	1.50	1.11	1.04	0.50	1.00			
	Cys	-	-	1.50	5.00	1.00	3.00	-	7.00	7.50*	4.00	11.00	-	-	7.33*	6.00	1.00				
	Gly	1.61	2.10*	1.27	1.06	-	6.00*	2.60*	2.06	2.09	2.00	5.00*	1.57	2.00	1.18	1.00					
	Ala	2.05*	2.89*	2.76*	1.94	8.00	1.59	1.59	1.83*	1.14	2.06	5.00*	1.18	1.63	1.00						
	His	1.57	1.89	0.86	1.15	4.00	4.33	1.20	1.70	0.91	2.29	3.44*	1.67	1.00							
	Met	1.73	1.76	1.31	2.33	3.00	2.20*	1.00	2.28*	1.55	1.81	2.50	1.00								
	Tyr	0.35	0.55	0.47	0.33	1.07	0.75	0.52	0.65	0.30+	0.87	1.00									
	Phe	0.53	0.88	1.12	0.42	1.48	1.04	0.58	0.94	0.70	1.00										
	Val	1.15	2.05*	0.80	1.00	4.50	1.57*	0.94	1.44*	1.00											
	Leu	0.62	1.35*	1.04	0.61	1.80	1.32*	1.03	1.00												
	Pro	1.00	1.05	0.62	0.86	1.50	1.08	1.00													
	Ile	0.66	0.84	0.76	0.53	3.25*	1.00														
	Trp	0.38	1.00	0.50	1.50	1.00															
	Asp	2.21	1.74*	1.21	1.00																
	Glu	0.97	1.61*	1.00																	
	Lys	0.91	1.00																		
	Arg	1.00																			

Each cell in the table is the ratio of replacements for a particular pair of amino acid. * indicates the replacements favored in the mesophilic to thermophilic direction whereas + indicates the opposite direction of such replacements have significant directional bias (*P *< 0.01).

**Table 4:** Major trends in synonymous codon and amino acid usage, as obtained from COA of RSCU and amino acid usage in genes/gene-products encoded by N. equitans

	Codon usage			Amino acid usage		
		
Principal axes	Variability explained (%)	Source of variation	Correlation coefficient* (r-value)	Variability explained (%)	Source of variation	Correlation coefficient* (r-value)
1^st ^axis	7.53	CAI	0.42	21.25	Mean Aromaticity	-0.66
		N_C_	-0.29		Gravy score	-0.56
		A_3S_	0.24		CAI	0.39
2^nd ^axis	7.05	_	_	13.32	Aliphatic Index	0.78
					Gravy Score	0.75
3^rd ^axis	5.71	_	_	11.12	MMW	0.80
					CAI	-0.31

* significant at p < 0.0001

**Table 5:** Comparison of amino acid usage between two clusters of probable membrane associated proteins and between potential highly and lowly expressed genes

	*N. equitans*							
	
Amino acids	Upper cluster genes		Lower cluster genes		Highly expressed genes		Lowly expressed genes	
		
	Number	(%)	Number	(%)	Number	(%)	Number	(%)
Phe	771	7.88*	649	4.20	367	3.55	402	5.20
Leu	1708	17.46*	1394	9.01	954	9.24	912	11.81
Ser	626	6.40	1060	6.85	383	3.71	409	5.30
Tyr	585	5.98	1117	7.22+	429	4.15	494	6.40
Cys	33	0.34	424	2.74+	46	0.45	85	1.10
Trp	102	1.04	137	0.89	95	0.92	77	1.00
Pro	329	3.36	597	3.86	488	4.72	314	4.07
His	96	0.98	118	0.76	155	**1.50**	61	0.79
Gln	155	1.58	347	2.24+	243	2.35	137	1.77
Arg	178	1.82	345	2.23	444	**4.30**	204	2.64
Ile	1373	14.04*	1604	10.37	866	8.38	940	12.17
Met	190	1.94*	168	1.09	226	2.19	128	1.66
Thr	426	4.35	879	5.68+	484	4.69	311	4.03
Asn	405	4.14	1384	8.95+	446	4.32	459	5.94
Lys	627	6.41	1504	9.72+	1099	10.64	840	10.88
Val	571	5.84	864	5.59	836	**8.09**	389	5.04
Ala	558	5.70*	600	3.88	703	**6.81**	344	4.45
Asp	267	2.73	724	4.68+	516	5.00	327	4.23
Glu	341	3.49	816	5.28+	895	**8.66**	508	6.58
Gly	441	4.51	737	4.76	655	**6.34**	383	4.96

Values marked with *or + are significantly (p < 0.001) more frequent in upper or lower cluster gene products respectively and the values in bold faces indicate significant (p < 0.001) overrepresentation of corresponding amino acids in potential highly expressed genes.

**Table 6:** Variation in synonymous codon usage in N. equitans and seven other obligatory host- associated microbial organisms

Organism	Accession No.	GC %	No. of tRNA genes	No. of rRNA operons	ORFs under study	Variation explained by COA on RSCU (%)	
						
						Axis1	Axis2
*Buchnera aphidicola*	AE013218	26.0	30	1	506	7.21	5.92
*Helicobacter hepaticus*	AE017125	38.0	36	1	1630	7.46	6.41
*Mycoplasma pulmonis*	AL445566	26.6	28	1	705	6.96	5.97
*Nanoarchaeum equitans*	AE017199	31.6	38	1*	487	7.53	7.05
*Rickettsia prowazekii*	AJ235269	29.0	32	1	772	6.68	5.72
*Ureaplasma parvum serovar3*	AF222894	25.5	29	2	558	7.59	7.05
*Wigglesworthia glossinidia*	BA000021	22.5	34	2	575	6.42	6.16
*Wolbachia pipientis*	AE017196	35.2	34	1	876	6.14	5.87

* the rRNAs are not in a single operon
