# Supplementary material for: Analysis of Nanoarchaeum equitans genome and proteome composition: indications for hyperthermophilic and parasitic adaptation
Source: BMC Genomics. 2006 Jul 25;7:186. doi: 10.1186/1471-2164-7-186 (PMC1574309; doi:10.1186/1471-2164-7-186)
Supplement: Additional file 1 — Amino acid replacement values between N. equitans proteins and their mesophilic orthologs. [file 1471-2164-7-186-S1.doc]

### Additional file 1. Amino acid replacement values between *N. equitans* proteins and their mesophilic orthologs

| *N. equitans* | | | | | | | | | | | | | | | | | | | | | |
| --- | --- | --- | --- | --- | --- | --- | --- | --- | --- | --- | --- | --- | --- | --- | --- | --- | --- | --- | --- | --- | --- |
|  |  | Arg | Lys | Glu | Asp | Trp | Ile | Pro | Leu | Val | Phe | Tyr | Met | His | Ala | Gly | Cys | Thr | Asn | Gln | Ser |
| 7 Mesophilic organisms | Ser | 47 | 133 | 141 | 87 | 9 | 46 | 61 | 46 | 56 | 20 | 28 | 22 | 13 | 179 | 69 | 2 | 107 | 74 | 41 | 415 |
|  | Gln | 44 | 112 | 113 | 30 | 3 | 27 | 24 | 34 | 14 | 8 | 26 | 17 | 12 | 23 | 11 | 0 | 11 | 28 | 251 | 25 |
|  | Asn | 53 | 139 | 100 | 106 | 2 | 24 | 10 | 25 | 15 | 7 | 39 | 12 | 34 | 20 | 60 | 2 | 37 | 408 | 24 | 47 |
|  | Thr | 40 | 124 | 82 | 32 | 8 | 89 | 40 | 68 | 110 | 16 | 40 | 16 | 12 | 90 | 25 | 6 | 573 | 48 | 38 | 103 |
|  | Cys | 5 | 3 | 3 | 5 | 1 | 12 | 2 | 14 | 45 | 8 | 11 | 2 | 3 | 44 | 6 | 99 | 12 | 3 | 0 | 14 |
|  | Gly | 37 | 105 | 66 | 50 | 6 | 24 | 39 | 33 | 23 | 12 | 15 | 11 | 10 | 112 | 1531 | 1 | 24 | 77 | 20 | 61 |
|  | Ala | 43 | 133 | 116 | 31 | 8 | 100 | 62 | 95 | 148 | 35 | 55 | 26 | 13 | 978 | 95 | 6 | 81 | 37 | 32 | 133 |
|  | His | 22 | 51 | 19 | 15 | 4 | 13 | 6 | 17 | 10 | 16 | 55 | 5 | 285 | 8 | 5 | 0 | 8 | 30 | 10 | 16 |
|  | Met | 19 | 44 | 21 | 14 | 3 | 108 | 6 | 169 | 48 | 29 | 30 | 217 | 3 | 22 | 7 | 0 | 21 | 6 | 14 | 9 |
|  | Tyr | 12 | 31 | 26 | 8 | 29 | 49 | 13 | 55 | 16 | 107 | 497 | 12 | 16 | 11 | 3 | 1 | 8 | 13 | 10 | 11 |
|  | Phe | 8 | 21 | 19 | 5 | 31 | 79 | 7 | 117 | 38 | 454 | 123 | 16 | 7 | 17 | 6 | 2 | 11 | 13 | 8 | 6 |
|  | Val | 23 | 78 | 48 | 11 | 9 | 603 | 30 | 244 | 916 | 54 | 54 | 31 | 11 | 130 | 11 | 6 | 75 | 23 | 19 | 23 |
|  | Leu | 31 | 111 | 49 | 11 | 18 | 474 | 30 | 1262 | 170 | 125 | 84 | 74 | 10 | 52 | 16 | 2 | 25 | 22 | 16 | 20 |
|  | Pro | 21 | 66 | 40 | 25 | 3 | 26 | 891 | 29 | 32 | 12 | 25 | 6 | 5 | 39 | 15 | 0 | 17 | 11 | 11 | 23 |
|  | Ile | 27 | 66 | 52 | 9 | 13 | 1113 | 24 | 359 | 385 | 76 | 65 | 49 | 3 | 63 | 4 | 4 | 40 | 14 | 7 | 15 |
|  | Trp | 5 | 7 | 3 | 3 | 134 | 4 | 2 | 10 | 2 | 21 | 27 | 1 | 1 | 1 | 0 | 1 | 2 | 1 | 2 | 0 |
|  | Asp | 31 | 155 | 285 | 781 | 2 | 17 | 29 | 18 | 11 | 12 | 24 | 6 | 13 | 16 | 47 | 1 | 32 | 110 | 40 | 43 |
|  | Glu | 83 | 365 | 1201 | 235 | 6 | 68 | 65 | 47 | 60 | 17 | 55 | 16 | 22 | 42 | 52 | 2 | 43 | 115 | 110 | 57 |
|  | Lys | 280 | 1166 | 227 | 89 | 7 | 79 | 63 | 82 | 38 | 24 | 56 | 25 | 27 | 46 | 50 | 0 | 52 | 83 | 72 | 47 |
|  | Arg | 825 | 307 | 86 | 14 | 13 | 41 | 21 | 50 | 20 | 15 | 34 | 11 | 14 | 21 | 23 | 0 | 30 | 52 | 29 | 35 |
|  | | | | | | | | | | | | | | | | | | | | | |

The value in each cell indicates the number of times the amino acid residues for the row was found in mesophilic orthologous proteins and was replaced by the amino acid for the column in the corresponding *N. equitans* protein.
