# Supplementary material for: Analysis of Nanoarchaeum equitans genome and proteome composition: indications for hyperthermophilic and parasitic adaptation
Source: BMC Genomics. 2006 Jul 25;7:186. doi: 10.1186/1471-2164-7-186 (PMC1574309; doi:10.1186/1471-2164-7-186)
Supplement: Additional file 2 — Synonymous codon usage in N. equitans [file 1471-2164-7-186-S2.doc]

### Additional file 2. Synonymous codon usage in *N. equitans*

| Amino Acid | Codon | Total | | High | | Low | | Amino Acid | Codon | Total | | High | | Low | |
| --- | --- | --- | --- | --- | --- | --- | --- | --- | --- | --- | --- | --- | --- | --- | --- |
|  |  | N | RSCU | N | RSCU | N | RSCU |  |  | N | RSCU | N | RSCU | N | RSCU |
|  | | | |  | |  |  |  | | | |  |  |  | |
| Phe | UUU | 4545 | 1.38 | 105 | 0.97 | 145 | 1.41 | Ser | UCU | 2411 | 2.1 | 84 | 2.27 | 77 | 2.12 |
|  | UUC* | 2049 | 0.62 | 111 | 1.03 | 60 | 0.59 |  | UCC* | 764 | 0.67 | 50 | 1.35 | 20 | 0.55 |
| Leu | UUA* | 7781 | 3.02 | 331 | 3.54 | 236 | 2.82 |  | UCA* | 751 | 0.65 | 30 | 0.81 | 16 | 0.44 |
|  | UUG | 3566 | 1.38 | 100 | 1.07 | 125 | 1.49 |  | UCG | 440 | 0.38 | 0 | 0.00 | 18 | 0.50 |
|  | | | |  | |  |  |  | | | |  |  |  | |
| Leu | CUU | 949 | 0.37 | 15 | 0.16 | 36 | 0.43 | Pro | CCU | 1856 | 1.25 | 62 | 0.78 | 62 | 1.44 |
|  | CUC | 247 | 0.1 | 4 | 0.04 | 13 | 0.16 |  | CCC | 1077 | 0.73 | 34 | 0.43 | 41 | 0.95 |
|  | CUA | 2732 | 1.06 | 110 | 1.18 | 85 | 1.02 |  | CCA* | 2783 | 1.87 | 220 | 2.77 | 55 | 1.28 |
|  | CUG | 174 | 0.07 | 1 | 0.01 | 7 | 0.08 |  | CCG | 225 | 0.15 | 2 | 0.03 | 14 | 0.33 |
|  | | | |  | |  |  |  | | | |  |  |  | |
| Ile | AUU | 4504 | 0.87 | 123 | 0.72 | 174 | 1.00 | Thr | ACU | 2319 | 1.53 | 105 | 1.47 | 71 | 1.47 |
|  | AUC | 1111 | 0.22 | 28 | 0.16 | 33 | 0.19 |  | ACC | 1060 | 0.7 | 53 | 0.74 | 40 | 0.83 |
|  | AUA* | 9882 | 1.91 | 364 | 2.12 | 316 | 1.81 |  | ACA | 2452 | 1.61 | 121 | 1.69 | 71 | 1.47 |
| Met | AUG | 2387 | 1 | 146 | 1.00 | 73 | 1.00 |  | ACG | 248 | 0.16 | 7 | 0.10 | 11 | 0.23 |
|  | | | |  | |  |  |  | | | |  |  |  | |
| Val | GUU | 3912 | 1.79 | 160 | 1.35 | 116 | 2.17 | Ala | GCU | 3012 | 1.58 | 188 | 1.62 | 81 | 1.62 |
|  | GUC | 424 | 0.19 | 24 | 0.20 | 10 | 0.19 |  | GCC | 1160 | 0.61 | 42 | 0.36 | 29 | 0.58 |
|  | GUA* | 3675 | 1.68 | 269 | 2.27 | 70 | 1.31 |  | GCA | 2706 | 1.42 | 196 | 1.69 | 72 | 1.44 |
|  | GUG | 749 | 0.34 | 22 | 0.19 | 18 | 0.34 |  | GCG | 766 | 0.4 | 39 | 0.34 | 18 | 0.36 |
|  | | | |  |  |  |  |  | | | |  |  |  | |
| Tyr | UAU | 6560 | 1.56 | 155 | 1.35 | 238 | 1.65 | Cys | UGU* | 759 | 1.29 | 28 | 1.60 | 27 | 1.17 |
|  | UAC* | 1830 | 0.44 | 74 | 0.65 | 51 | 0.35 |  | UGC | 419 | 0.71 | 7 | 0.40 | 19 | 0.83 |
| ter | UAA | 272 | 0 | 14 | 1.75 | 18 | 2.25 | ter | UGA | 105 | 0 | 6 | 0.75 | 1 | 0.13 |
| ter | UAG | 110 | 0 | 4 | 0.50 | 5 | 0.63 | Trp | UGG | 1440 | 1 | 65 | 1.00 | 39 | 1.00 |
|  | | | |  |  |  |  |  | | | |  |  |  | |
| His | CAU | 1650 | 1.66 | 78 | 1.47 | 32 | 1.60 | Arg | CGU | 105 | 0.11 | 0 | 0 | 10 | 0.58 |
|  | CAC | 336 | 0.34 | 28 | 0.53 | 8 | 0.40 |  | CGC | 88 | 0.09 | 0 | 0 | 13 | 0.75 |
| Gln | CAA* | 3036 | 1.87 | 145 | 1.93 | 70 | 1.75 |  | CGA | 131 | 0.14 | 0 | 0 | 19 | 1.10 |
|  | CAG | 211 | 0.13 | 5 | 0.07 | 10 | 0.25 |  | CGG | 31 | 0.03 | 0 | 0 | 8 | 0.46 |
|  | | | |  |  |  |  |  | | | |  |  |  | |
| Asn | AAU | 5199 | 1.33 | 127 | 1.02 | 171 | 1.34 | Ser | AGU | 1166 | 1.01 | 17 | 0.46 | 35 | 0.96 |
|  | AAC* | 2639 | 0.67 | 122 | 0.98 | 85 | 0.66 |  | AGC | 1361 | 1.18 | 41 | 1.11 | 52 | 1.43 |
| Lys | AAA | 13145 | 1.66 | 510 | 1.58 | 431 | 1.67 | Arg | AGA* | 3594 | 3.79 | 216 | 4.21 | 35 | 2.02 |
|  | AAG | 2737 | 0.34 | 134 | 0.42 | 85 | 0.33 |  | AGG* | 1743 | 1.84 | 92 | 1.79 | 19 | 1.10 |
|  | | | |  |  |  |  |  | | | |  |  |  | |
| Asp | GAU | 5675 | 1.54 | 190 | 1.35 | 137 | 1.49 | Gly | GGU* | 2291 | 1.17 | 142 | 1.49 | 64 | 1.07 |
|  | GAC | 1691 | 0.46 | 91 | 0.65 | 47 | 0.51 |  | GGC | 1550 | 0.79 | 54 | 0.57 | 58 | 0.97 |
| Glu | GAA* | 8727 | 1.51 | 451 | 1.60 | 219 | 1.41 |  | GGA* | 2470 | 1.26 | 138 | 1.45 | 65 | 1.08 |
|  | GAG | 2805 | 0.49 | 111 | 0.40 | 92 | 0.59 |  | GGG | 1537 | 0.78 | 47 | 0.49 | 53 | 0.88 |
|  | | | |  | |  |  |  | | | |  |  |  | |

* indicates corresponding codons are significantly overrepresented (p < 0.05) among putatively highly expressed genes.
