# Supplementary figures and images for: Analysis of Nanoarchaeum equitans genome and proteome composition: indications for hyperthermophilic and parasitic adaptation
Source: BMC Genomics. 2006 Jul 25;7:186. doi: 10.1186/1471-2164-7-186 (PMC1574309; doi:10.1186/1471-2164-7-186)

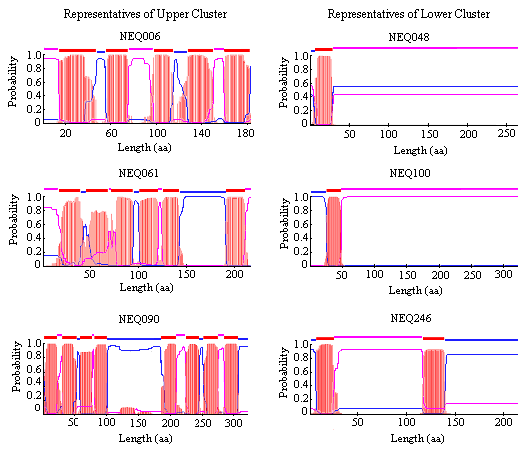

Supplement: Additional file 3 — TMHMM plot of the probable membrane associated proteins encoded by genes taken from upper and lower clusters generated by COA on amino acid usage for N. equitans genome. The plots of the left panel are the representatives of upper cluster and those in the right panels are the representatives of lower cluster. Red, blue and pink colours indicate the transmembrane, outside and inside regions of the protein respectively. [file 1471-2164-7-186-S3.tiff]
